# Supplementary material for: Multiplicity of the Agrobacterium Infection of Nicotiana benthamiana for Transient DNA Delivery
Source: ACS Synth Biol. 2023 Aug 9;12(8):2329–38. doi: 10.1021/acssynbio.3c00148 (PMC10443027; doi:10.1021/acssynbio.3c00148)
Supplement: Supplementary file 1 — sb3c00148_si_001.pdf [file sb3c00148_si_001.pdf]

Multiplicity of *Agrobacterium* infection of *Nicotiana benthamiana* for transient DNA delivery

Erik D. Carlson[1], Jakub Rajniak[2], & Elizabeth S. Sattely[1,3,#]

Department of Chemical Engineering[1], Department of Bioengineering[2], Howard Hughes Medical Institute[3], Stanford University, Stanford, California 94305, USA.
[#]Corresponding author, sattely@stanford.edu

Supporting Information

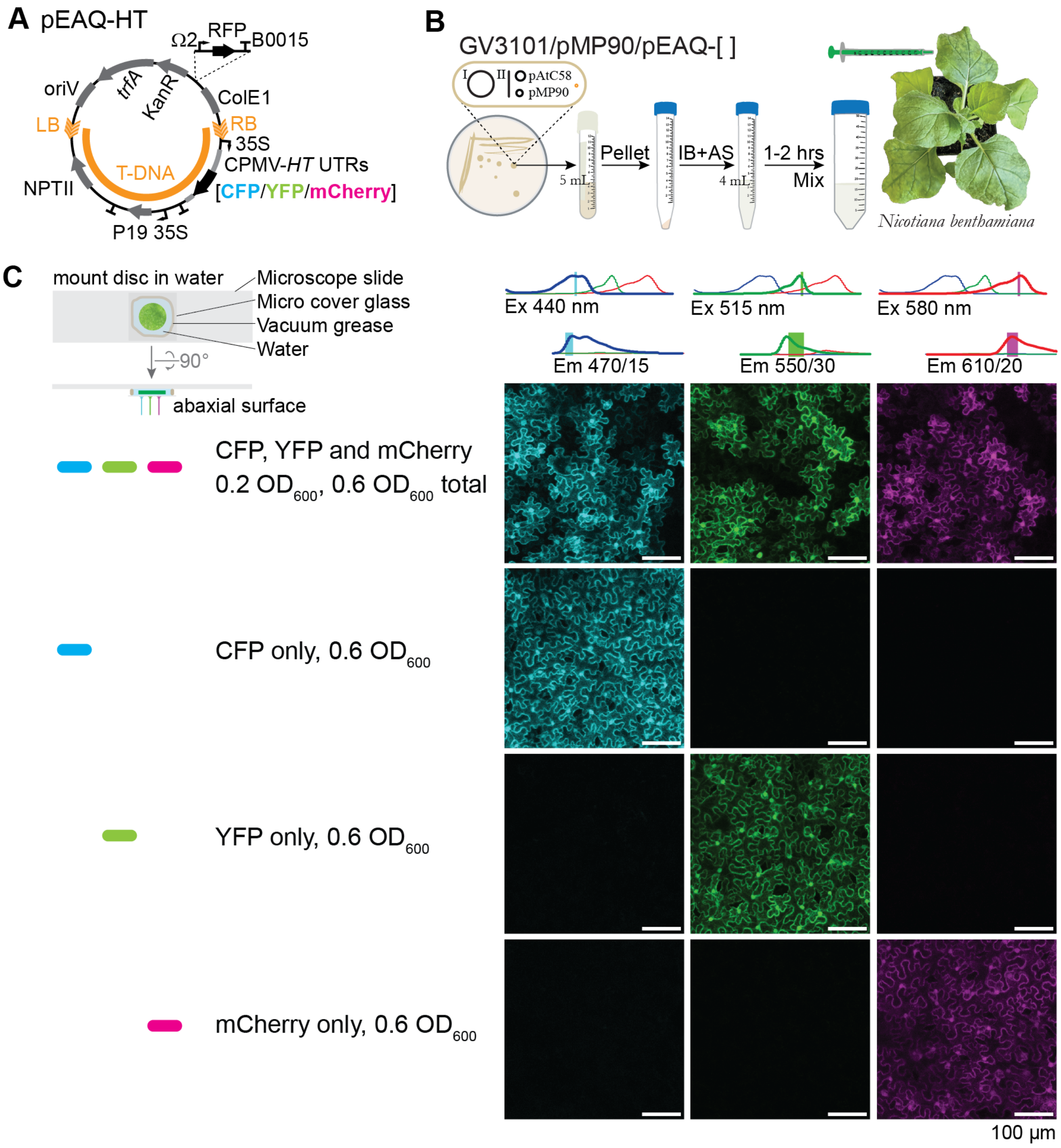

Supplementary Figure 1. *Agrobacterium*-mediated transformation for transient heterologous protein expression and confocal microscopy imaging. **A.** pEAQ-HT expression vector map. **B.** *Agrobacterium*-mediated transient expression infiltration method. IB: induction buffer, AS: acetosyringone. **C.** Confocal microscopy slide preparation set up and imaging settings. Confocal microscope images of underside of leaf, 5 days post-infiltration, 0.2 OD_{600} per strain CFP/YFP/mCherry, 0.6 OD_{600} total, and individual strain infiltrations at 0.6 OD_{600}. Excitation (Ex) and emission (Em) settings indicated above images. Emission filter settings [midpoint]/[spread] in nm. For example: 610/20 is a 600-620 nm filter.

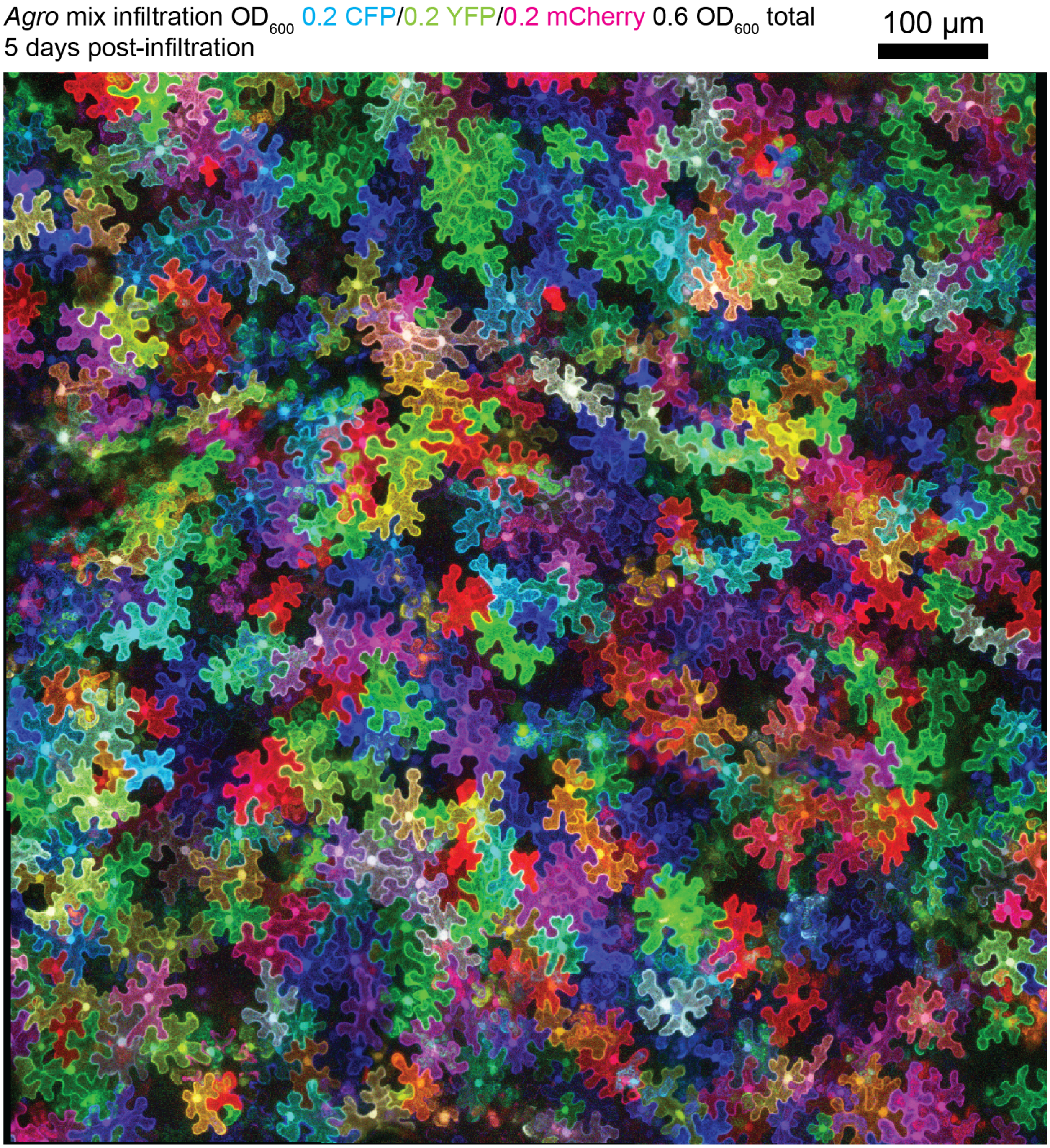

Supplementary Figure 2. Confocal microscope image of underside of leaf, 5 days post-infiltration, 0.2 OD_{600} per strain CFP/YFP/mCherry, 0.6 OD_{600} total. Tilescan image 3x3, stitched together using ImageJ.

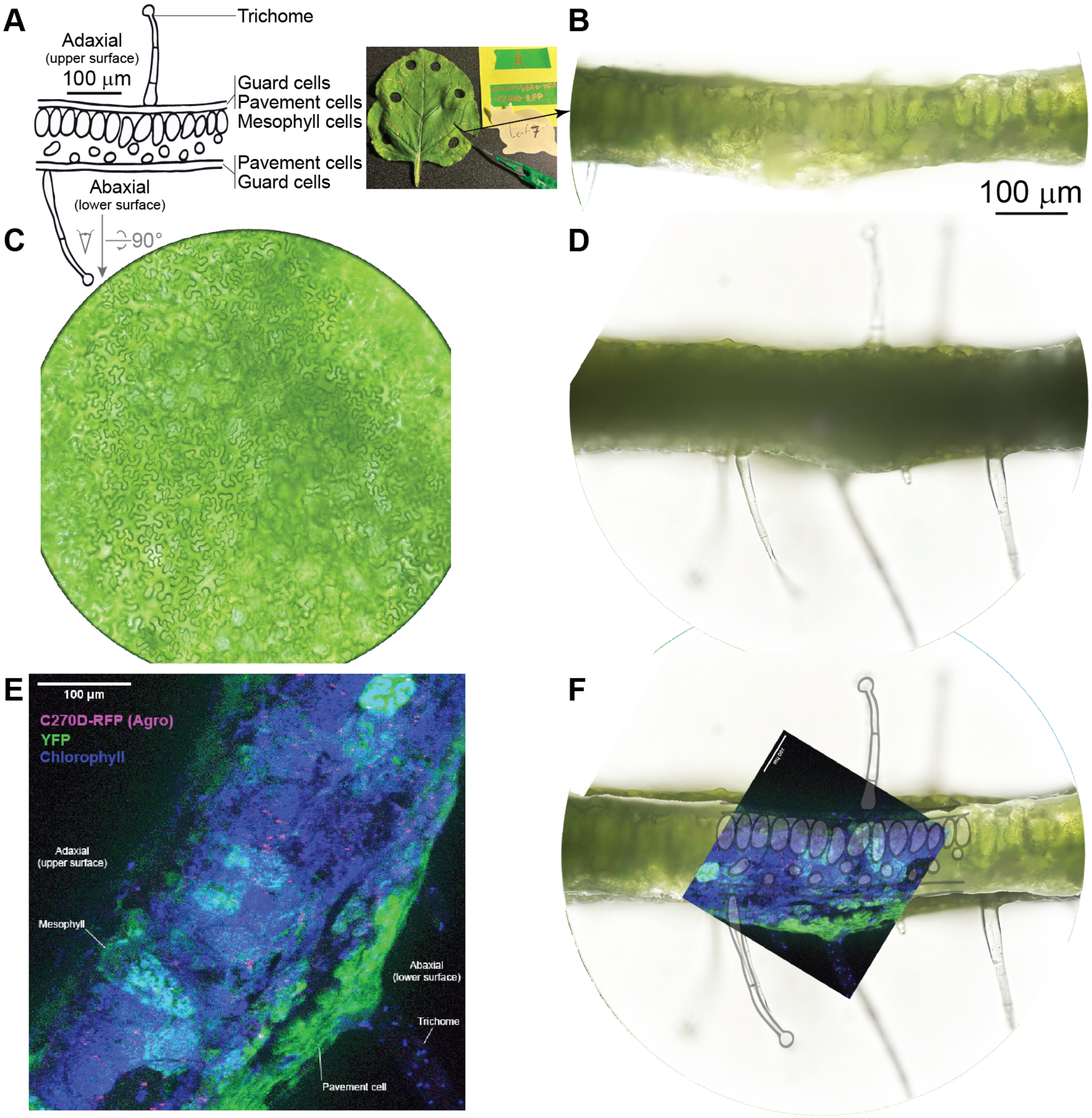

Supplementary Figure 3. Brightfield and confocal microscopy images used to construct *N. benthamiana* leaf cross section diagram. **A.** Thin cross section cut from a 5 dpi infiltration spot, infiltrated with GV3101(pMP90)/pEAQ-YFP-RFP, with fresh scalpel. **B.** Brightfield image of leaf cross-section, focused on cut surface. **C.** Bright field image of Abaxial (lower) surface of leaf. **D.** Bright field image of leaf cross-section, focused further into tissue from cut surface. **E.** Confocal microscopy image of RFP in magenta (*Agrobacterium*), YFP T-DNA product in green, and chlorophyll autofluorescence in blue. **F.** Overlays of images in panels **B**, **D** and **E** with diagram trace from panel **A**.

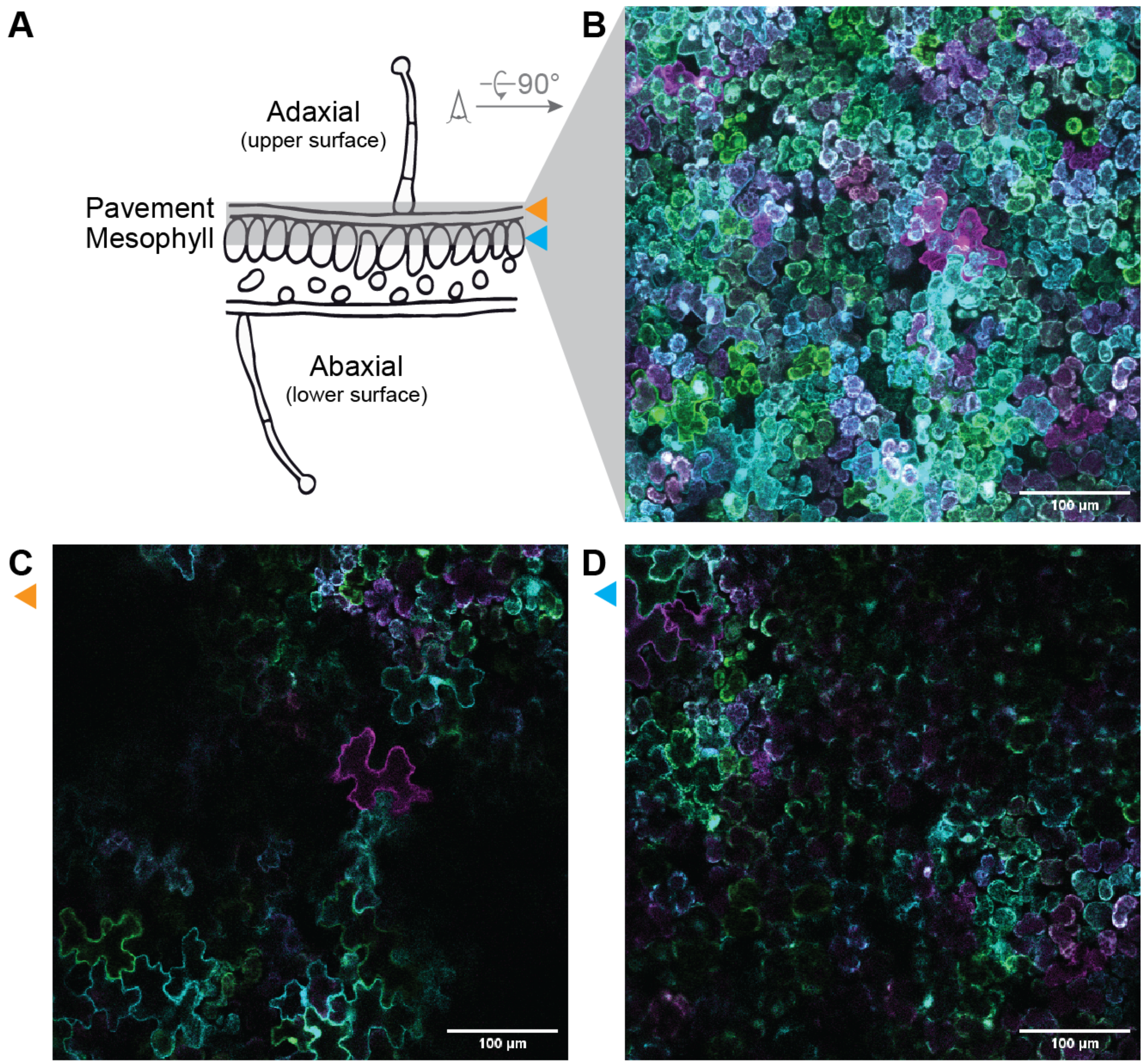

Supplementary Figure 4. Confocal microscopy of upper surface showing transient expression in mesophyll cells. **A**. Cross section diagram of leaf. Gray box indicates full z stack max projection in (**B**). Orange arrow indicates z slice across pavement cell layer (**C**). Blue arrow indicates z slice across mesophyll cell layer (**D**). White scale bars indicate 100 µm.

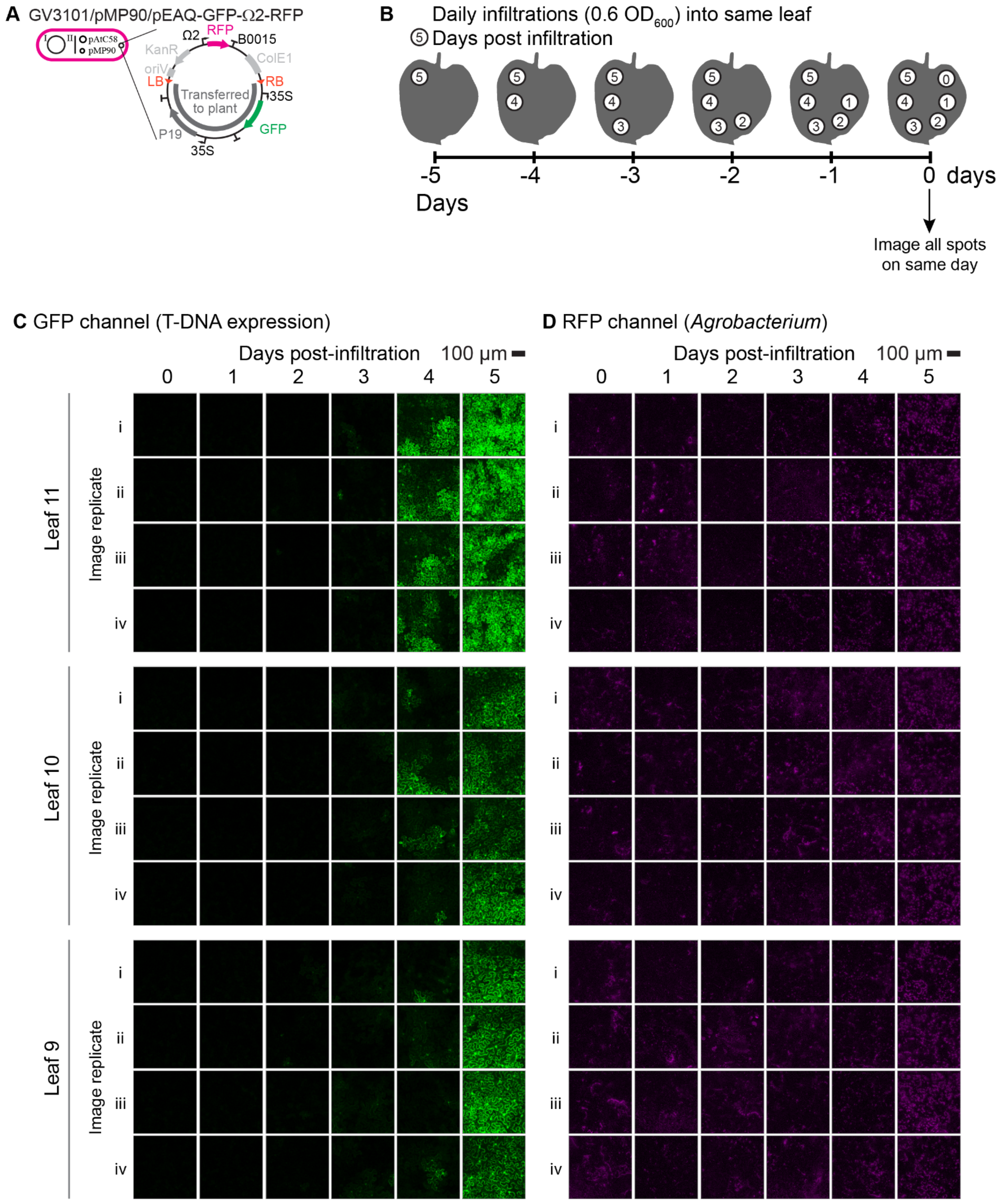

Supplementary Figure 5. Tracking of RFP-labeled GV3101 strain with a GFP T-DNA load at 0.6 infiltration OD_{600}. **A.** GV3101/pMP90/pEAQ-GFP-RFP strain. **B.** Infiltration scheme. Daily infiltrations into same leaf, and image on the same day. **C.** GFP channel images. **D.** RFP channel images.

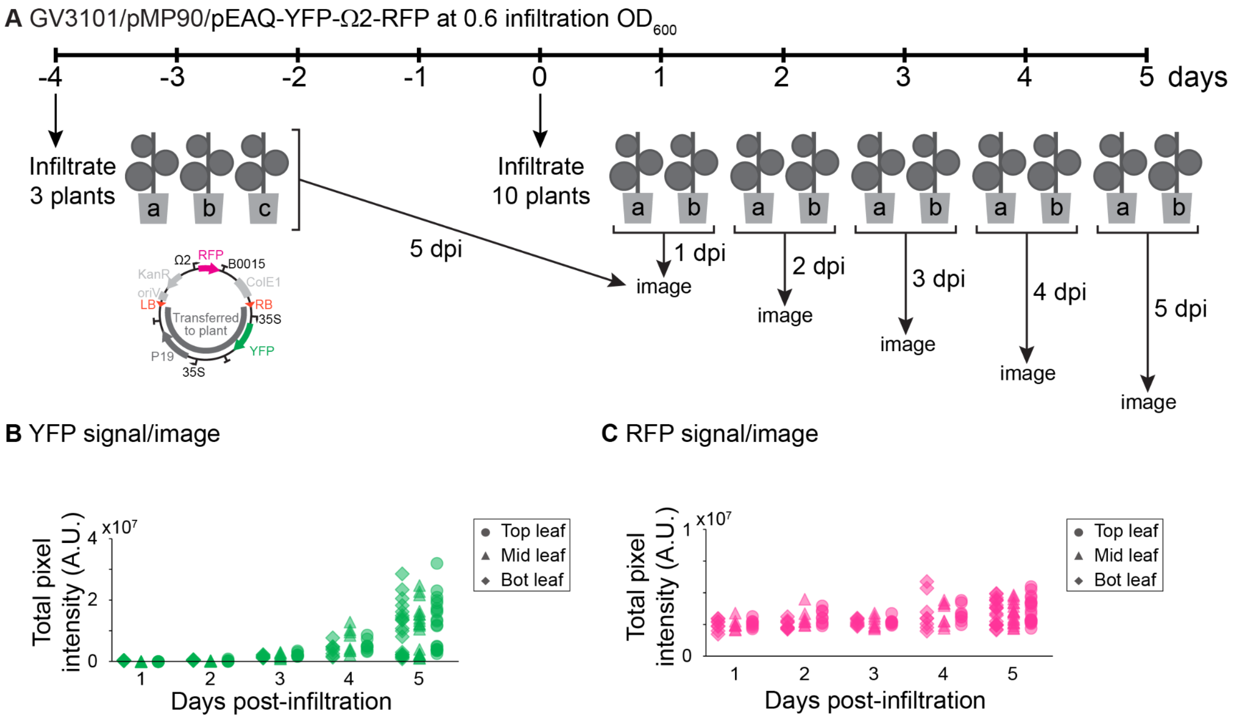

Supplementary Figure 6. A. Tracking of RFP-labeled GV3101 strain with a YFP T-DNA load at 0.6 infiltration OD_{600}, into top 3 fully expanded leaves of 6-week old *Nicotiana benthamiana* plants. Infiltrations done in two batches, and imaged on sequential days using the same imaging laser setting and levels. **B.** YFP channel. **D.** RFP channel.

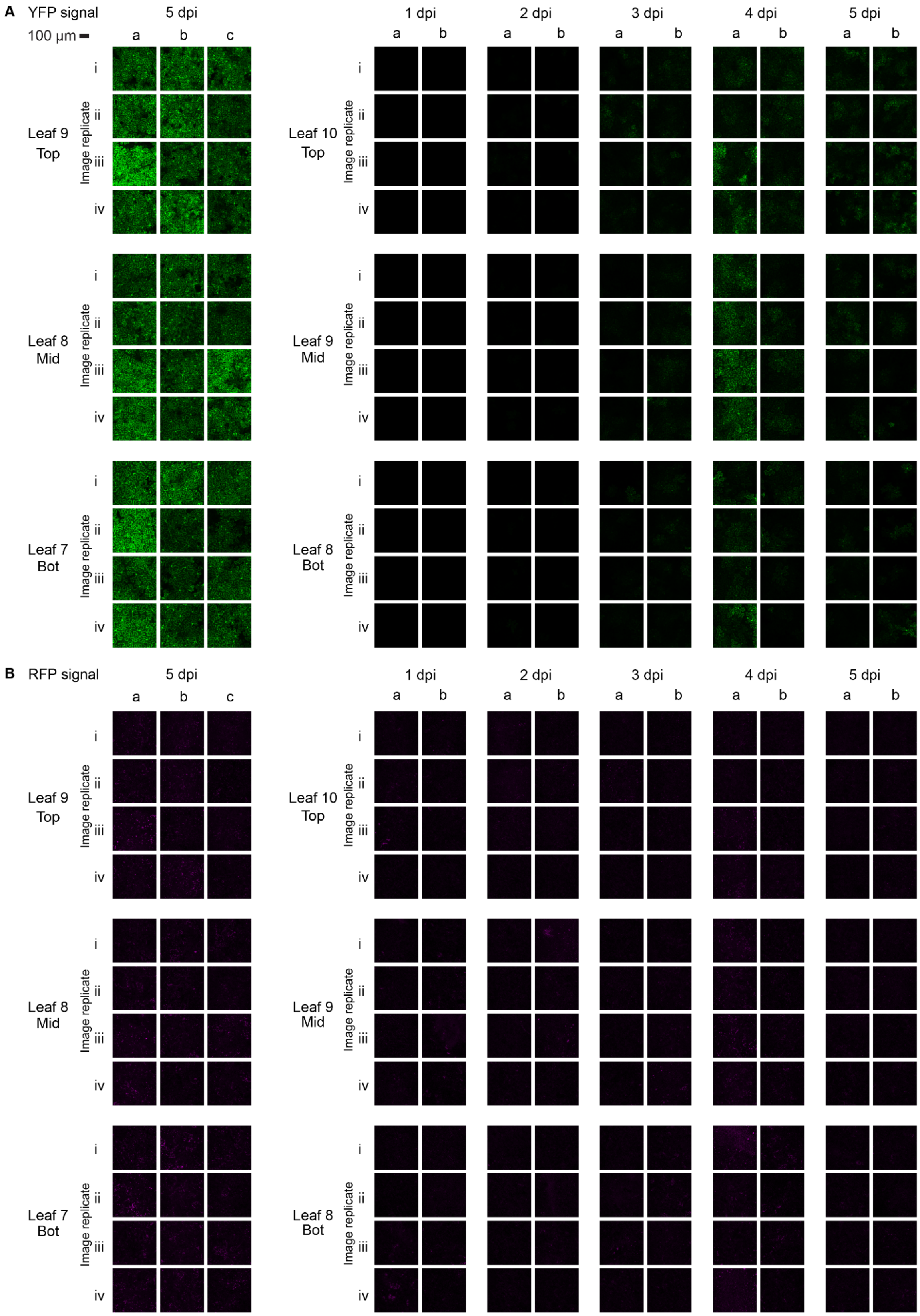

Supplementary Figure 7. Raw microscopy images for Supplementary Figure 6 experiment. **A.** YFP channel images. **B.** RFP channel images.

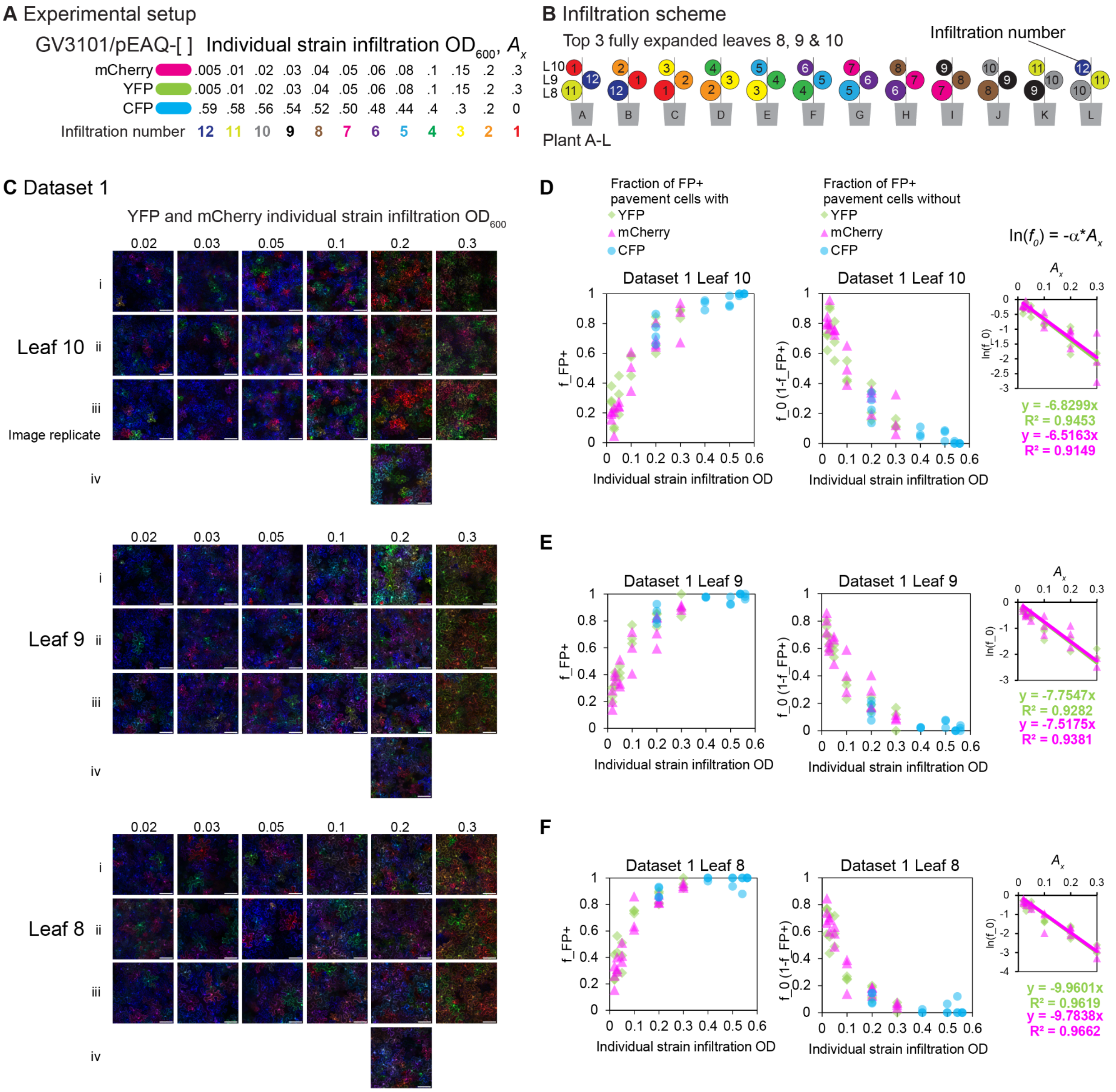

Supplementary Figure 8. Multiplicity of infection determination, Dataset 1. **A,B.** Experimental setup. **A.** *Agrobacterium* strain co-infiltration mixes **B.** Infiltration scheme into the top 3 fully expanded leaves. **C.** Overview of microscopy image data collected for Dataset 1. **D-F.** Determining α values for Leaf 10 (**D**), Leaf 9 (**E**) and Leaf 8 (**F**).

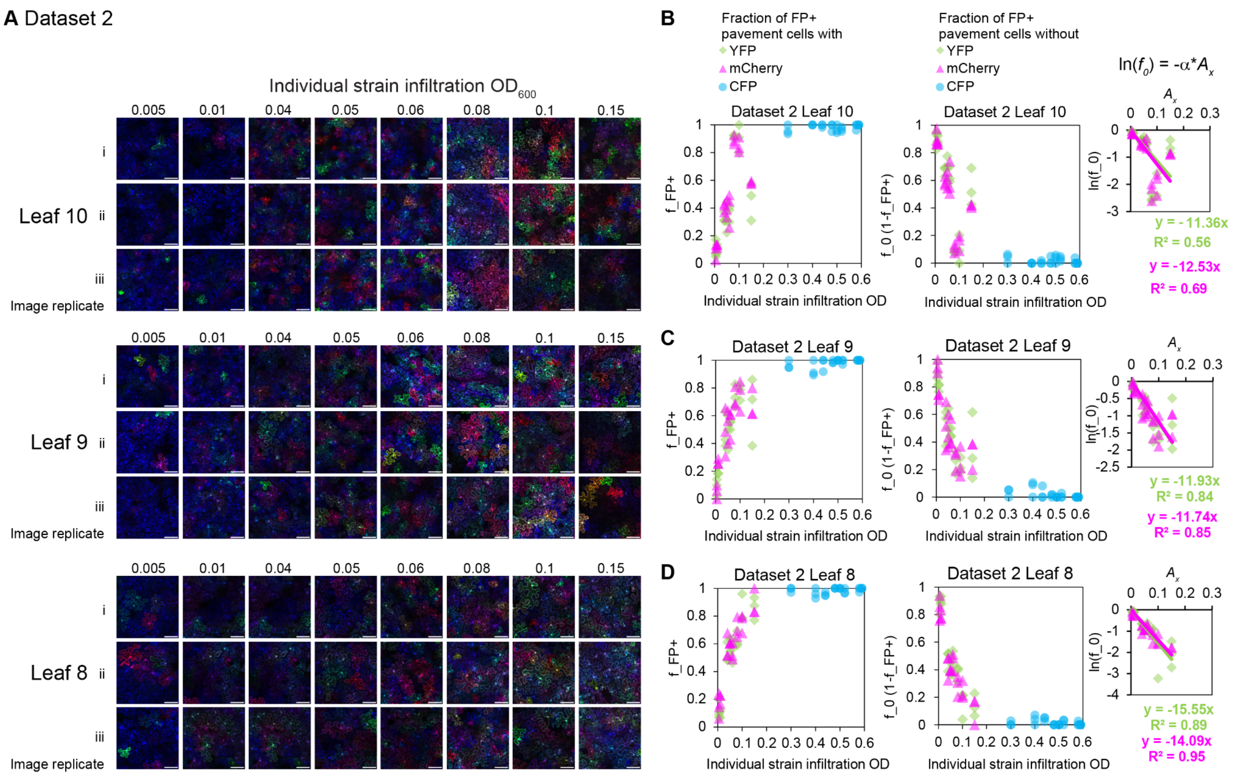

Supplementary Figure 9. Multiplicity of infection determination, Dataset 2. **A.** Overview of microscopy image data collected for Dataset 1. **B-D.** Determining α values for Leaf 10 (**B**), Leaf 9 (**C**) and Leaf 8 (**D**).

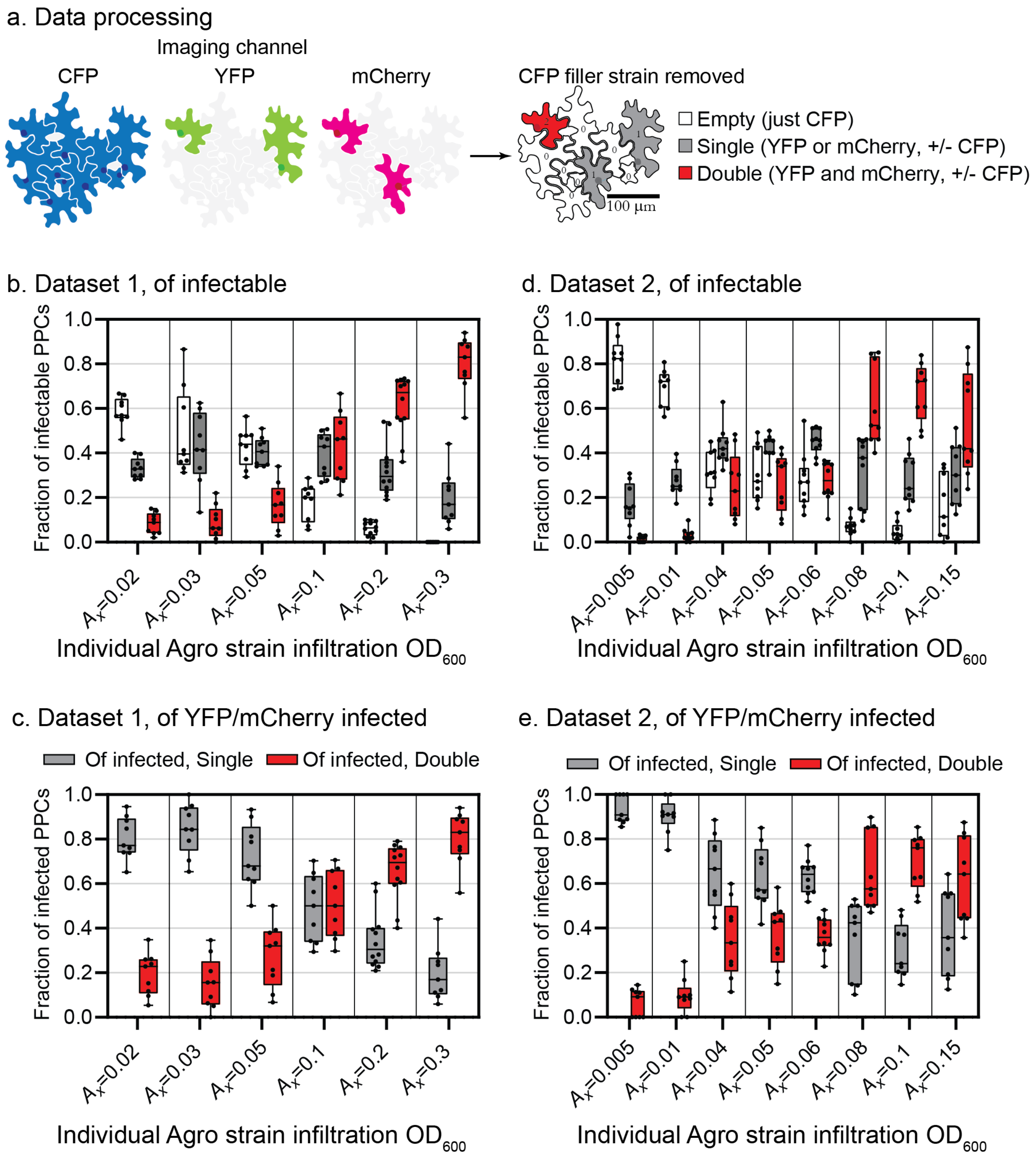

Supplementary Figure 10. A. Experimental data processed for YFP and mCherry indicator strains, empty, single and double infection bins. **B, D**. Fraction of infectable plant pavement cells (PPCs) with no YFP or mCherry (empty), YFP or mCherry (single), and YFP and mCherry (double) for Dataset 1 (**B**) and Dataset 2 (**D**). **C, E**. Data processed for only indicator strain positive pavement cells (YFP and/or mCherry) for Dataset 1 (**C**) and Dataset 2 (**E**).

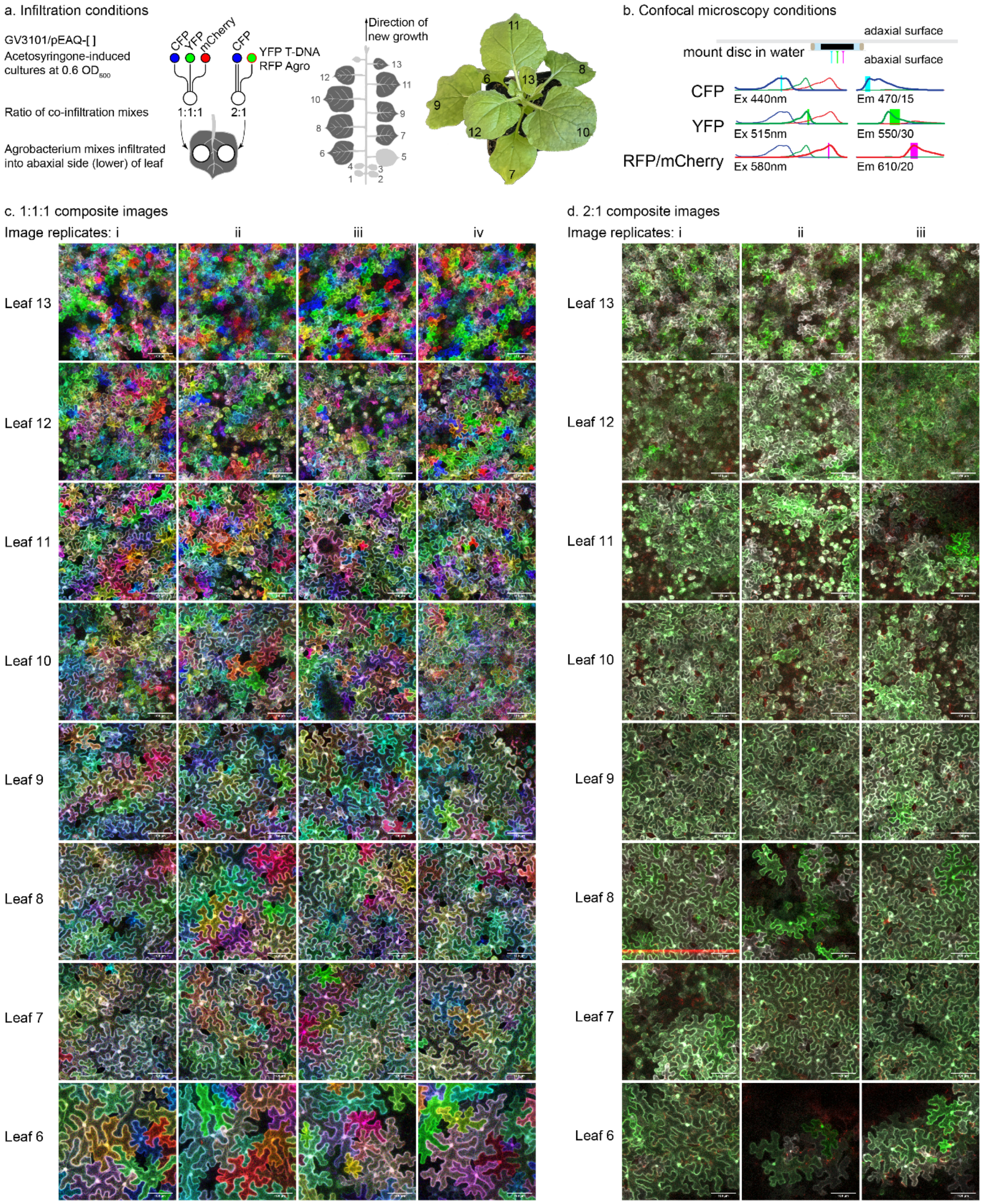

Supplementary Figure 11. Microscopy imaging dataset for Figure 4. **A.** Infiltration scheme of Acetosyringone-induced culture mixes. On left side of leaf, 1:1:1 mix of CFP:YFP:mCherry cultures, each at a final OD_{600} of 0.2, total infiltration OD_{600} 0.6. On right side of leaf, a 2:1 mix of CFP strain (0.4 OD_{600}) with a YFP (T-DNA) and RFP (*Agro* tag) strain (0.2 OD_{600}). Mixes were infiltrated into leaves 6-13 on a 6-week old *N. benthamiana* plant. **B.** Confocal microscopy imaging conditions. **C**. Microscopy dataset for 1:1:1 CFP:YFP:mCherry infiltration spot, 4 image replicates per leaf. Composite image shown with CFP channel colored blue, YFP channel colored green, and mCherry channel colored red. White scale bar indicates 100 μm. **D**. Microscopy dataset for 2:1 CFP:YFP-RFP infiltration spot.

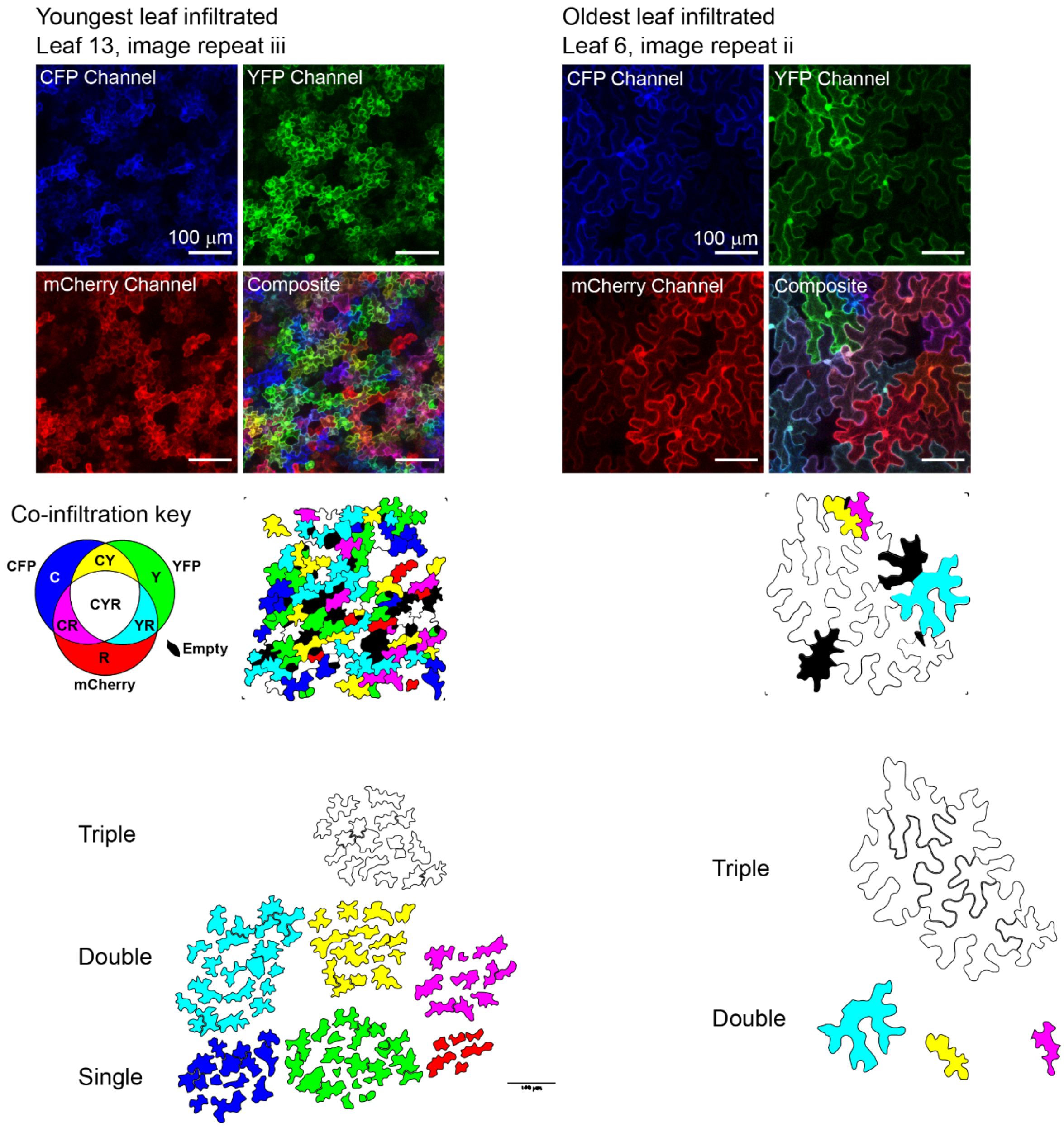

Supplementary Figure 12. Image processing and quantification procedure and example. Representative traced and counted images. Co-expression key: C: CFP, Y: YFP, R: mCherry. A white plant pavement cell indicates co-infection by all 3 strains, black, no detectable fluorescent protein. White scale bar 100 µm.

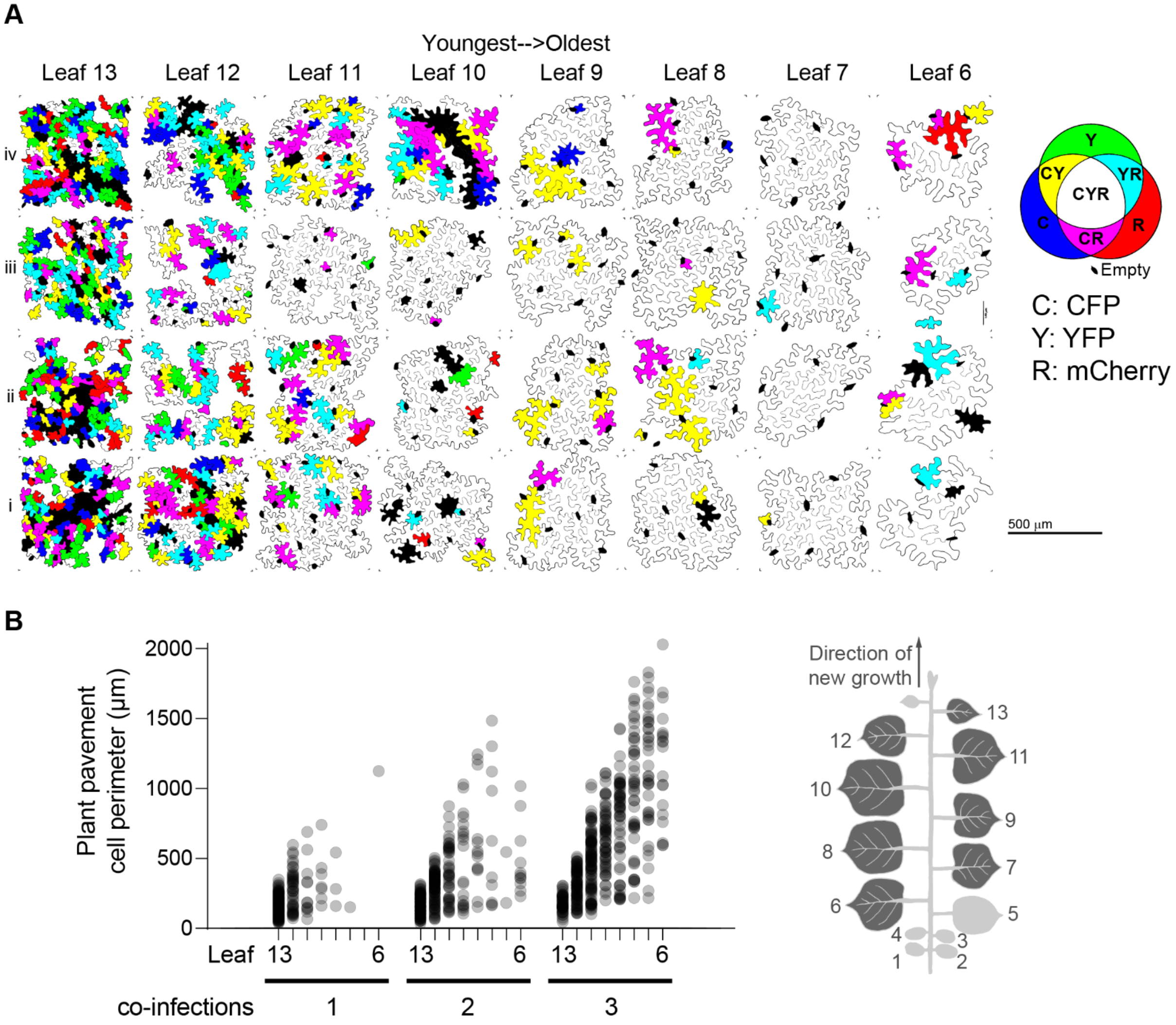

Supplementary Figure 13. Analysis of data from 1:1:1 CFP:YFP:mCherry strain co-infiltration, 0.2 OD_{600} per strain, total infiltration OD_{600} = 0.6. **A.** Plant pavement cell traces for perimeter quantification and co-infection binning. C: CFP, Y: YFP, R: RFP. **B**. Co-infections and pavement cell perimeters by leaf.

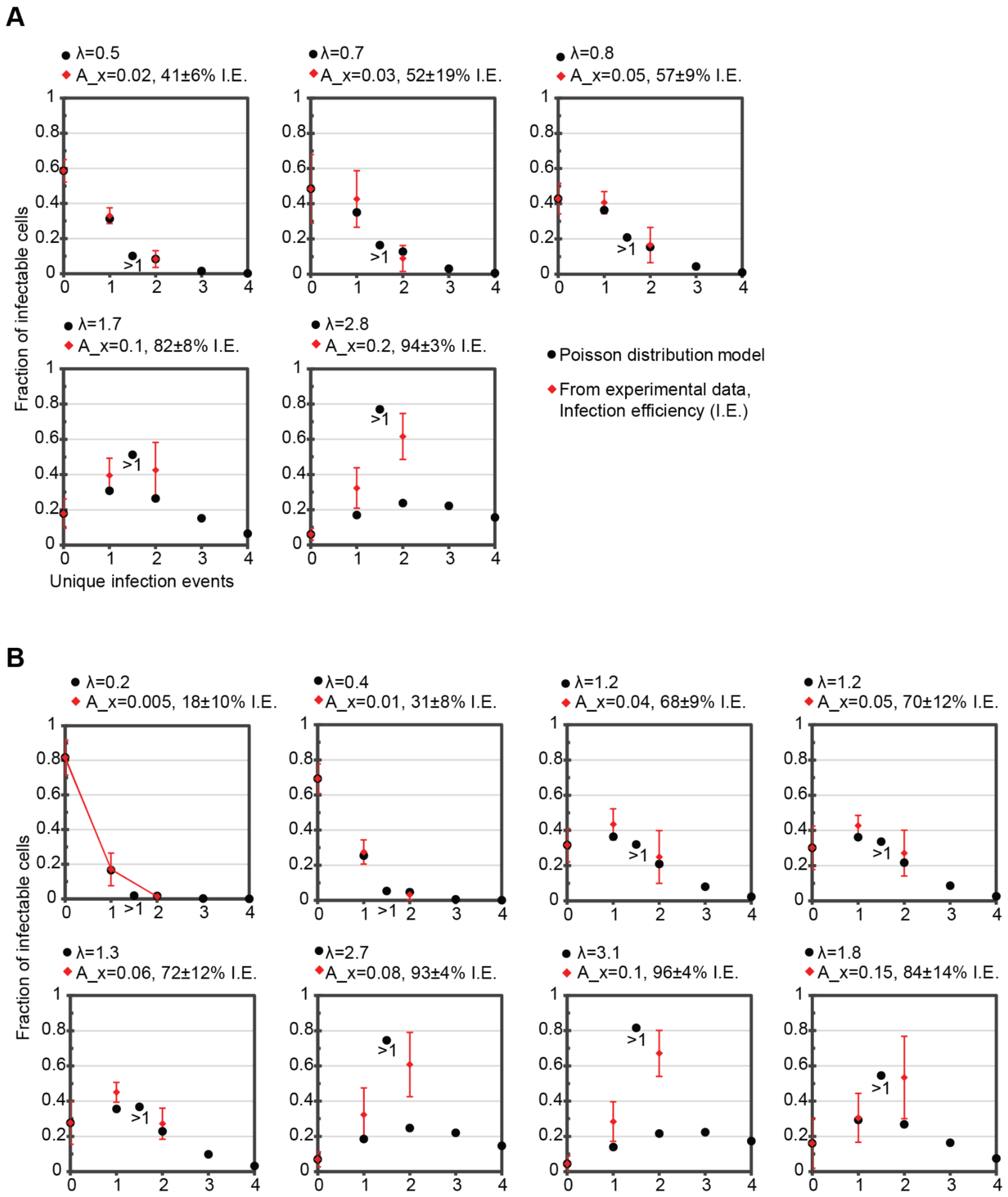

Supplementary Figure 14. Expected number of infection events per plant pavement cell at various infection efficiencies based on Poisson distribution (equation 1). Curves with solid connection lines are based on the Poisson distribution form, with overlaid comparison of experimental data in red square with dashed line. Datasets 1 (**A**) and 2 (**B**).

Supplemental Table 1. Data for figure 3, Dataset 1.

		mCherry	
		+	-
YFP	+		
	-		

Dataset--Infiltration condition_plant_leaf_repeat Leaf number	Image edge (µm)	Individual Agro strain infiltration OD_{600}, A_x: CFP OD_{600} (A_{CFP})	YFP OD_{600} (A_{YFP})	mCherry OD_{600} ($A_{mCherry}$)	Number of FP+ pavement cells per image: CFP single	YFP single	mCherry single	CFP/YFP	CFP/mCherry	YFP/mCherry	CFP/YFP/mCherry	FP- pavement cells (estimated)	FP- pairs of guard cells	Guard cells	FP+ pavement cells	FP+ and FP- pavement cells	Total plant cells (FP+, FP- pavement cells, guard cells)					
Dataset 1–10 J top i redo Leaf 10	465	0.56	0.02	0.02	27	0	0	11	8	0	2	6	16	54	48	54	108	ACTUAL		EXPECTED		CHISQ.TEST
Dataset 1–10 J top ii Leaf 10	465	0.56	0.02	0.02	33	0	0	16	3	0	6	7	24	65	58	65	130	13	38	9	42	0.115
Dataset 1–10_J_top iii Leaf 10	465	0.56	0.02	0.02	35	0	0	11	6	0	5	10	14	67	57	67	134	17	95	21	91	
Dataset 1–9 I top i Leaf 10	465	0.54	0.03	0.03	58	0	0	6	3	0	0	5	28	72	67	72	144	ACTUAL		EXPECTED		CHISQ.TEST
Dataset 1–9 I top ii Leaf 10	465	0.54	0.03	0.03	49	0	0	13	14	0	5	4	30	85	81	85	170	6	25	5	26	0.664
Dataset 1–9 I top iii Leaf 10	465	0.54	0.03	0.03	48	1	0	5	13	0	1	3	22	71	68	71	142	30	155	31	154	
Dataset 1–7_G_top i Leaf 10	465	0.5	0.05	0.05	37	3	3	18	12	0	4	7	22	84	77	84	168	ACTUAL		EXPECTED		CHISQ.TEST
Dataset 1–7_G_top ii Leaf 10	465	0.5	0.05	0.05	25	5	0	14	7	0	7	10	16	68	58	68	136	13	51	16	48	0.251
Dataset 1–7 G top iii Leaf 10	465	0.5	0.05	0.05	39	1	0	10	17	0	2	12	20	81	69	81	162	39	101	36	104	
Dataset 1–4_D_top i Leaf 10	465	0.4	0.1	0.1	19	1	0	23	9	2	12	10	16	76	66	76	152	ACTUAL		EXPECTED		CHISQ.TEST
Dataset 1–4_D_top ii Leaf 10	465	0.4	0.1	0.1	15	1	2	11	21	1	18	6	32	75	69	75	150	54	52	52	54	0.555
Dataset 1–4_D_top iii Leaf 10	465	0.4	0.1	0.1	15	3	1	13	10	3	18	7	26	70	63	70	140	43	49	45	47	
Dataset 1–2 B top i Leaf 10	582	0.2	0.2	0.2	3	1	2	10	10	7	40	7	32	80	73	80	160	ACTUAL		EXPECTED		CHISQ.TEST
Dataset 1–2 B top ii Leaf 10	465	0.2	0.2	0.2	3	0	5	12	9	6	14	6	20	55	49	55	110	110	48	113	45	0.335
Dataset 1–2 B top iii Leaf 10	465	0.2	0.2	0.2	5	7	1	5	14	9	9	6	18	56	50	56	112	45	14	42	17	
Dataset 1–2 B top iv Leaf 10	465	0.2	0.2	0.2	3	6	0	7	4	7	18	8	14	53	45	53	106					
Dataset 1–1_A_top i Leaf 10	465	0	0.3	0.3	0	6	8	0	0	35	0	11	14	60	49	60	120	ACTUAL		EXPECTED		CHISQ.TEST
Dataset 1–1_A_top ii Leaf 10	465	0	0.3	0.3	0	17	6	0	0	29	0	11	20	63	52	63	126	118	27	121	24	0.031
Dataset 1–1 A top iii Leaf 10	465	0	0.3	0.3	0	4	7	0	0	54	0	5	22	70	65	70	140	21	0	18	3	
Dataset 1–10_K_mid i Leaf 9	465	0.56	0.02	0.02	33	0	1	10	4	0	2	8	20	52	50	58	110	ACTUAL		EXPECTED		CHISQ.TEST
Dataset 1–10_K_mid ii Leaf 9	465	0.56	0.02	0.02	32	1	1	8	8	0	1	4	18	56	51	55	111	11	27	8	30	0.153
Dataset 1–10_K_mid iii Leaf 9	465	0.56	0.02	0.02	30	0	0	8	7	0	8	5	28	48	53	58	106	21	95	24	92	
Dataset 1–9 J mid i Leaf 9	465	0.54	0.03	0.03	19	0	0	9	10	0	8	5	14	54	46	51	105	ACTUAL		EXPECTED		CHISQ.TEST
Dataset 1–9 J mid ii Leaf 9	465	0.54	0.03	0.03	16	0	0	15	12	0	5	4	22	49	48	52	101	19	33	20	32	0.683
Dataset 1–9 J mid iii Leaf 9	465	0.54	0.03	0.03	19	0	0	9	14	0	6	4	20	43	48	52	95	36	54	35	55	
Dataset 1–7_H_mid i Leaf 9	465	0.5	0.05	0.05	19	1	3	14	8	0	6	5	20	47	51	56	103	ACTUAL		EXPECTED		CHISQ.TEST
Dataset 1–7_H_mid ii Leaf 9	465	0.5	0.05	0.05	12	0	3	8	7	0	11	7	18	54	41	48	102	25	33	22	36	0.282
Dataset 1–7_H_mid iii Leaf 9	465	0.5	0.05	0.05	23	0	0	10	7	1	7	6	14	49	48	54	103	28	54	31	51	
Dataset 1–4_E_mid i Leaf 9	465	0.4	0.1	0.1	12	0	0	17	6	1	13	0	24	52	49	49	101	ACTUAL		EXPECTED		CHISQ.TEST
Dataset 1–4_E_mid ii Leaf 9	465	0.4	0.1	0.1	4	0	0	7	5	1	22	4	16	54	39	43	97	58	33	51	40	0.012
Dataset 1–4_E_mid iii Leaf 9	465	0.4	0.1	0.1	9	0	0	9	6	1	20	2	18	41	45	47	88	17	25	24	18	
Dataset 1–2 C mid i Leaf 9	465	0.2	0.2	0.2	4	0	2	4	3	4	30	7	20	34	47	54	88	ACTUAL		EXPECTED		CHISQ.TEST
Dataset 1–2 C mid ii Leaf 9	465	0.2	0.2	0.2	4	5	1	8	1	2	21	7	20	35	42	49	84	109	30	103	36	0.007
Dataset 1–2 C mid iii Leaf 9	465	0.2	0.2	0.2	2	0	1	5	3	2	27	12	18	47	40	52	99	17	14	23	8	
Dataset 1–2 C mid iv Leaf 9	465	0.2	0.2	0.2	4	3	3	5	3	3	20	13	10	51	41	54	105					
Dataset 1–1_B_mid i Leaf 9	465	0	0.3	0.3	0	3	6	0	0	27	0	5	16	53	36	41	94	ACTUAL		EXPECTED		CHISQ.TEST
Dataset 1–1_B_mid ii Leaf 9	465	0	0.3	0.3	0	3	0	0	0	29	0	2	22	30	32	34	64	82	10	83	9	0.272
Dataset 1–1 B mid iii Leaf 9	465	0	0.3	0.3	0	4	4	0	0	26	0	1	20	36	34	35	71	10	0	9	1	
Dataset 1–10_L_bot i Leaf 8	465	0.56	0.02	0.02	26	0	0	7	4	0	2	8	22	43	39	47	90	ACTUAL		EXPECTED		CHISQ.TEST
Dataset 1–10_L_bot ii Leaf 8	465	0.56	0.02	0.02	23	0	0	14	6	0	7	1	20	44	50	51	95	15	27	11	31	0.076
Dataset 1–10_L_bot iii Leaf 8	465	0.56	0.02	0.02	28	0	0	6	11	0	6	2	28	55	51	53	108	21	77	25	73	
Dataset 1–9 K bot i Leaf 8	465	0.54	0.03	0.03	9	1	2	5	7	0	1	5	4	44	25	30	74	ACTUAL		EXPECTED		CHISQ.TEST
Dataset 1–9 K bot ii Leaf 8	465	0.54	0.03	0.03	10	0	0	12	8	0	2	4	12	51	32	36	87	12	32	14	30	0.305
Dataset 1–9 K bot iii Leaf 8	465	0.54	0.03	0.03	15	0	0	14	3	0	9	3	14	48	41	44	92	20	34	18	36	
Dataset 1–7_I_bot i Leaf 8	465	0.5	0.05	0.05	15	1	2	7	6	0	16	4	24	42	47	51	93	ACTUAL		EXPECTED		CHISQ.TEST
Dataset 1–7_I_bot ii Leaf 8	465	0.5	0.05	0.05	22	0	0	5	11	0	8	0	22	31	46	46	77	33	21	23	31	0.001
Dataset 1–7_I_bot iii Leaf 8	465	0.5	0.05	0.05	18	0	0	8	6	0	9	2	20	39	41	43	82	25	55	35	45	
Dataset 1–4_F_bot i Leaf 8	465	0.4	0.1	0.1	2	0	0	3	7	0	24	1	18	36	36	37	73	ACTUAL		EXPECTED		CHISQ.TEST
Dataset 1–4_F_bot ii Leaf 8	465	0.4	0.1	0.1	8	0	0	8	3	0	22	2	24	48	41	43	91	65	23	61	27	0.077
Dataset 1–4_F_bot iii Leaf 8	465	0.4	0.1	0.1	3	1	0	11	7	0	19	3	14	28	41	44	72	17	13	21	9	
Dataset 1–2 D bot i Leaf 8	465	0.2	0.2	0.2	4	1	1	4	4	5	29	7	18	36	48	55	91	ACTUAL		EXPECTED		CHISQ.TEST
Dataset 1–2 D bot ii Leaf 8	465	0.2	0.2	0.2	1	1	2	3	5	0	28	4	22	44	40	44	88	130	24	128	26	0.392
Dataset 1–2 D bot iii Leaf 8	465	0.2	0.2	0.2	1	1	4	7	0	2	34	2	34	68	49	51	119	21	6	23	4	
Dataset 1–2 D bot iv Leaf 8	465	0.2	0.2	0.2	0	1	1	6	4	1	31	4	22	44	44	48	92					
Dataset 1–1_C_bot i Leaf 8	465	0	0.3	0.3	0	3	2	0	0	36	0	1	24	48	41	42	90	ACTUAL		EXPECTED		CHISQ.TEST
Dataset 1–1_C_bot ii Leaf 8	465	0	0.3	0.3	0	1	2	0	0	24	0	4	14	28	27	31	59	92	6	92	6	0.610
Dataset 1–1_C_bot iii Leaf 8	465	0	0.3	0.3	0	2	0	0	0	32	0	5	16	32	34	39	71	4	0	4	0	

Supplemental Table 2. Data for figure 3, Dataset 2

			mCherry	
			+	-
YFP	+			
	-			

		Individual Agro strain infiltration OD_{600}, A_x			Number of FP+ pavement cells per image																	
Dataset--Infiltration condition_plant_leaf_repeat Leaf number	Image edge (µm)	CFP OD_{600} A_{CFP}	YFP OD_{600} A_{YFP}	mCherry OD_{600} $A_{mCherry}$	CFP single	YFP single	mCherry single	CFP/YFP	CFP/mCherry	YFP/mCherry	CFP/YFP/mCherry	FP- pavement cells (estimated)	FP- pairs of guard cells	Guard cells	FP+ pavement cells	FP+ and FP- pavement cells	Total plant cells (FP+, FP- pavement cells, guard cells)					
Dataset 2--12_H_top iii Leaf 10	465	0.59	0.005	0.005	36	0	0	9	7	0	0	1	26	52	52	53	105	ACTUAL		EXPECTED		CHISQ.TEST
Dataset 2--12_H_top ii Leaf 10	465	0.59	0.005	0.005	37	0	0	2	1	0	0	6	14	28	40	46	74	1	13	1	13	0.758
Dataset 2--12_H_top i Leaf 10	465	0.59	0.005	0.005	39	0	0	2	4	0	1	4	22	44	46	50	94	12	112	12	112	
Dataset 2--11_G_top iii Leaf 10	465	0.58	0.01	0.01	38	0	0	6	6	0	1	3	20	40	51	54	94	ACTUAL		EXPECTED		CHISQ.TEST
Dataset 2--11_G_top ii Leaf 10	465	0.58	0.01	0.01	42	0	2	3	4	0	1	4	24	48	52	56	104	2	15	2	15	0.874
Dataset 2--11_G_top i Leaf 10	465	0.58	0.01	0.01	39	0	0	6	6	0	0	6	16	32	51	57	89	18	119	18	119	
Dataset 2--8_F_top iii Leaf 10	465	0.52	0.04	0.04	18	1	1	17	20	1	4	2	26	52	62	64	116	ACTUAL		EXPECTED		CHISQ.TEST
Dataset 2--8_F_top ii Leaf 10	465	0.52	0.04	0.04	28	0	2	11	13	0	8	9	26	52	62	71	123	23	40	26	37	0.393
Dataset 2--8_F_top i Leaf 10	465	0.52	0.04	0.04	27	1	0	10	19	0	10	4	26	52	67	71	123	55	73	52	76	
Dataset 2--7_E_top iii Leaf 10	465	0.5	0.05	0.05	21	2	2	15	16	0	14	4	28	56	70	74	130	ACTUAL		EXPECTED		CHISQ.TEST
Dataset 2--7_E_top ii Leaf 10	465	0.5	0.05	0.05	33	0	0	8	19	0	7	3	30	60	67	70	130	27	41	27	41	0.923
Dataset 2--7_E_top i Leaf 10	465	0.5	0.05	0.05	32	0	3	16	15	0	6	13	18	36	72	85	121	55	86	55	86	
Dataset 2--6_D_top top iii Leaf 10	465	0.48	0.06	0.06	26	1	2	14	20	1	17	3	30	60	81	84	144	ACTUAL		EXPECTED		CHISQ.TEST
Dataset 2--6_D_top top ii Leaf 10	465	0.48	0.06	0.06	29	0	0	14	19	0	16	5	30	60	78	83	143	42	44	35	51	0.042
Dataset 2--6_D_top top i Leaf 10	465	0.48	0.06	0.06	42	0	0	15	12	0	8	6	26	52	77	83	135	53	97	60	90	
Dataset 2--5_C_top iii Leaf 10	465	0.44	0.08	0.08	3	0	0	3	2	0	44	1	22	44	52	53	97	ACTUAL		EXPECTED		CHISQ.TEST
Dataset 2--5_C_top ii Leaf 10	465	0.44	0.08	0.08	0	0	1	4	3	0	46	0	34	68	54	54	122	132	12	128	16	0.001
Dataset 2--5_C_top i Leaf 10	465	0.44	0.08	0.08	2	0	0	5	2	0	42	1	24	48	51	52	100	8	5	12	1	
Dataset 2--4_B_top iii Leaf 10	465	0.4	0.1	0.1	0	0	0	10	0	0	41	0	28	56	51	51	107	ACTUAL		EXPECTED		CHISQ.TEST
Dataset 2--4_B_top ii redo Leaf 10	465	0.4	0.1	0.1	1	0	0	4	4	0	47	2	30	60	56	58	118	127	18	123	22	0.008
Dataset 2--4_B_top i Leaf 10	465	0.4	0.1	0.1	5	0	0	4	6	0	39	1	28	56	54	55	111	10	6	14	2	
Dataset 2--3_A_top iii Leaf 10	465	0.3	0.15	0.15	15	0	0	3	7	0	18	3	14	28	43	46	74	ACTUAL		EXPECTED		CHISQ.TEST
Dataset 2--3_A_top ii Leaf 10	465	0.3	0.15	0.15	14	0	2	3	13	0	10	5	14	28	42	47	75	45	16	36	25	0.001
Dataset 2--3_A_top i Leaf 10	465	0.3	0.15	0.15	10	2	0	8	10	1	16	6	22	44	47	53	97	32	39	41	30	
Dataset 2--12_A_mid i Leaf 9	465	0.59	0.005	0.005	41	0	0	6	2	0	1	3	26	52	50	53	105	ACTUAL		EXPECTED		CHISQ.TEST
Dataset 2--12_A_mid ii Leaf 9	465	0.59	0.005	0.005	42	0	0	4	4	0	1	3	18	36	51	54	90	2	11	1	12	0.098
Dataset 2--12_A_mid iii Leaf 9	465	0.59	0.005	0.005	45	0	0	1	0	0	0	6	24	48	46	52	100	6	128	7	127	
Dataset 2--11_H_mid i Leaf 9	465	0.58	0.01	0.01	33	0	0	8	12	0	2	2	32	64	55	57	121	ACTUAL		EXPECTED		CHISQ.TEST
Dataset 2--11_H_mid ii Leaf 9	465	0.58	0.01	0.01	27	0	0	7	12	0	2	1	30	60	48	49	109	9	22	8	23	0.734
Dataset 2--11_H_mid iii Leaf 9	465	0.58	0.01	0.01	31	0	0	7	8	0	5	2	30	60	51	53	113	32	91	33	90	
Dataset 2--8_G_mid i Leaf 9	465	0.52	0.04	0.04	6	0	1	6	6	0	16	5	12	24	35	40	64	ACTUAL		EXPECTED		CHISQ.TEST
Dataset 2--8_G_mid ii Leaf 9	465	0.52	0.04	0.04	11	0	0	7	9	0	8	3	14	28	35	38	66	28	24	25	27	0.220
Dataset 2--8_G_mid iii Leaf 9	465	0.52	0.04	0.04	16	0	0	11	8	0	4	3	18	36	39	42	78	24	33	27	30	
Dataset 2--7_F_mid i Leaf 9	465	0.5	0.05	0.05	9	0	0	12	10	0	17	2	28	56	48	50	106	ACTUAL		EXPECTED		CHISQ.TEST
Dataset 2--7_F_mid ii Leaf 9	465	0.5	0.05	0.05	19	0	0	8	10	0	8	5	18	36	45	50	86	40	27	36	31	0.141
Dataset 2--7_F_mid iii Leaf 9	465	0.5	0.05	0.05	9	0	0	7	13	0	15	7	14	28	44	51	79	33	37	37	33	
Dataset 2--6_E_mid i Leaf 9	465	0.48	0.06	0.06	11	0	0	12	15	0	21	4	22	44	59	63	107	ACTUAL		EXPECTED		CHISQ.TEST
Dataset 2--6_E_mid ii Leaf 9	465	0.48	0.06	0.06	9	0	0	13	16	1	17	3	26	52	56	59	111	52	40	51	41	0.660
Dataset 2--6_E_mid iii Leaf 9	465	0.48	0.06	0.06	17	1	0	14	11	0	13	0	24	48	56	56	104	42	37	43	36	
Dataset 2--5_D_mid i Leaf 9	465	0.44	0.08	0.08	2	0	0	6	12	0	22	4	16	32	42	46	78	ACTUAL		EXPECTED		CHISQ.TEST
Dataset 2--5_D_mid ii Leaf 9	465	0.44	0.08	0.08	4	1	1	7	5	1	18	4	20	40	37	41	81	58	21	58	21	0.917
Dataset 2--5_D_mid iii Leaf 9	465	0.44	0.08	0.08	2	0	0	7	3	0	17	4	14	28	29	33	61	21	8	21	8	
Dataset 2--4_C_mid i Leaf 9	465	0.4	0.1	0.1	5	1	0	8	6	3	15	0	22	44	38	38	82	ACTUAL		EXPECTED		CHISQ.TEST
Dataset 2--4_C_mid ii Leaf 9	465	0.4	0.1	0.1	1	1	1	5	5	1	19	3	16	32	33	36	68	62	18	60	20	0.282
Dataset 2--4_C_mid iii Leaf 9	465	0.4	0.1	0.1	2	0	0	3	4	0	24	4	12	24	33	37	61	16	8	18	6	
Dataset 2--3_B_mid i Leaf 9	465	0.3	0.15	0.15	1	0	0	9	6	0	34	1	28	56	50	51	107	ACTUAL		EXPECTED		CHISQ.TEST
Dataset 2--3_B_mid ii Leaf 9	465	0.3	0.15	0.15	3	0	2	12	6	0	16	4	20	40	39	43	83	62	24	59	27	0.243
Dataset 2--3_B_mid iii Leaf 9	465	0.3	0.15	0.15	12	0	0	3	12	2	10	2	20	40	39	41	81	26	16	29	13	
Dataset 2--12_B_bot i Leaf 8	465	0.59	0.005	0.005	24	0	0	6	4	0	1	3	14	28	35	38	66	ACTUAL		EXPECTED		CHISQ.TEST
Dataset 2--12_B_bot ii Leaf 8	465	0.59	0.005	0.005	21	0	0	1	6	0	1	1	14	28	29	30	58	2	10	2	10	0.814
Dataset 2--12_B_bot iii Leaf 8	465	0.59	0.005	0.005	28	0	0	3	2	0	0	2	16	32	33	35	67	12	73	12	73	
Dataset 2--11_A_bot i Leaf 8	465	0.58	0.01	0.01	26	0	1	4	5	0	0	2	20	40	36	38	78	ACTUAL		EXPECTED		CHISQ.TEST
Dataset 2--11_A_bot ii Leaf 8	465	0.58	0.01	0.01	32	0	0	2	9	0	1	3	20	40	44	47	87	3	9	3	10	0.708
Dataset 2--11_A_bot iii Leaf 8	465	0.58	0.01	0.01	28	0	0	3	7	0	2	0	22	44	40	40	84	22	86	23	86	
Dataset 2--8_H_bot i Leaf 8	465	0.52	0.04	0.04	10	0	0	6	7	0	10	4	12	24	33	37	61	ACTUAL		EXPECTED		CHISQ.TEST
Dataset 2--8_H_bot ii Leaf 8	465	0.52	0.04	0.04	9	0	0	5	6	1	8	6	10	20	29	35	55	34	15	28	21	0.011
Dataset 2--8_H_bot iii Leaf 8	465	0.52	0.04	0.04	6	1	0	3	6	0	15	3	12	24	31	34	58	19	25	25	19	
Dataset 2--7_G_bot i Leaf 8	465	0.5	0.05	0.05	9	0	0	4	6	0	14	0	18	36	33	33	69	ACTUAL		EXPECTED		CHISQ.TEST
Dataset 2--7_G_bot ii Leaf 8	465	0.5	0.05	0.05	5	0	0	8	7	0	13	1	16	32	33	34	66	36	16	31	21	0.048
Dataset 2--7_G_bot iii Leaf 8	465	0.5	0.05	0.05	6	0	0	4	6	0	9	1	10	20	25	26	46	19	20	24	15	
Dataset 2--6_F_bot i Leaf 8	465	0.48	0.06	0.06	10	0	0	8	6	0	13	5	12	24	37	42	66	ACTUAL		EXPECTED		CHISQ.TEST
Dataset 2--6_F_bot ii Leaf 8	465	0.48	0.06	0.06	11	0	0	10	11	0	9	4	20	40	41	45	85	37	29	35	31	0.578
Dataset 2--6_F_bot iii Leaf 8	465	0.48	0.06	0.06	5	0	0	11	10	0	15	3	22	44	41	44	88	27	26	29	24	
Dataset 2--5_E_bot i Leaf 8	465	0.44	0.08	0.08	3	0	0	5	13	1	17	1	18	36	39	40	76	ACTUAL		EXPECTED		CHISQ.TEST
Dataset 2--5_E_bot ii Leaf 8	465	0.44	0.08	0.08	6	0	0	8	10	2	14	4	24	48	40	44	92	52	22	53	21	0.776
Dataset 2--5_E_bot iii Leaf 8	465	0.44	0.08	0.08	3	0	1	9	8	1	17	4	16	32	39	43	75	32	12	31	13	
Dataset 2--4_D_bot i Leaf 8	465	0.4	0.1	0.1	1	0	0	8	5	0	14	4	10	20	28	32	52	ACTUAL		EXPECTED		CHISQ.TEST
Dataset 2--4_D_bot ii Leaf 8	465	0.4	0.1	0.1	1	0	1	5	4	1	16	1	16	32	28	29	61	50	18	51	17	0.396
Dataset 2--4_D_bot iii Leaf 8	465	0.4	0.1	0.1	0	0	0	5	1	1	18	4	10	20	25	29	49	11	2	10	3	
Dataset 2--3_C_bot i Leaf 8	465	0.3	0.15	0.15	1	0	0	4	1	0	24	2	18	36	30	32	68	ACTUAL		EXPECTED		CHISQ.TEST
Dataset 2--3_C_bot ii Leaf 8	465	0.3	0.15	0.15	0	0	0	0	4	0	28	3	18	36	32	35	71	77	6	74	9	0.002
Dataset 2--3_C_bot iii Leaf 8	465	0.3	0.15	0.15	4	0	1	2	3	0	25	2	18	36	35	37	73	9	5	12	2	

Supplemental Table 3. Data for figure 3

	PPCs with 1 infection, n_1				PPCs with 2 infections, n_2				PPCs with 3 infections, n_3				Total infected PPCs				f_1, Fraction of infected w/1				f_2, Fraction of infected w/2				f_3, Fraction of infected w/3			
**Leaf\Image**	**i**	**ii**	**iii**	**iv**	**i**	**ii**	**iii**	**iv**	**i**	**ii**	**iii**	**iv**	**i**	**ii**	**iii**	**iv**	**i**	**ii**	**iii**	**iv**	**i**	**ii**	**iii**	**iv**	**i**	**ii**	**iii**	**iv**
**13**	42	54	42	57	39	30	46	39	19	7	17	11	100	91	105	107	0.4	0.6	0.4	0.5	0.4	0.3	0.4	0.4	0.2	0.1	0.2	0.1
**12**	13	10	6	15	28	27	17	18	17	16	27	15	58	53	50	48	0.2	0.2	0.1	0.3	0.5	0.5	0.3	0.4	0.3	0.3	0.5	0.3
**11**	1	4	1	6	13	13	2	16	31	20	24	22	45	37	27	44	0	0.1	0	0.1	0.3	0.4	0.1	0.4	0.7	0.5	0.9	0.5
**10**	1	3	0	3	4	2	3	11	20	15	18	6	25	20	21	20	0	0.2	0	0.2	0.2	0.1	0.1	0.6	0.8	0.8	0.9	0.3
**9**	0	0	0	3	2	5	3	3	15	14	15	12	17	19	18	18	0	0	0	0.2	0.1	0.3	0.2	0.2	0.9	0.7	0.8	0.7
**8**	0	0	0	1	1	5	2	2	13	8	12	11	14	13	14	14	0	0	0	0.1	0.1	0.4	0.1	0.1	0.9	0.6	0.9	0.8
**7**	0	0	0	0	1	0	1	0	10	7	11	9	11	7	12	9	0	0	0	0	0.1	0	0.1	0	0.9	1	0.9	1
**6**	0	0	0	1	1	3	3	4	5	6	4	4	6	9	7	9	0	0	0	0.1	0.2	0.3	0.4	0.4	0.8	0.7	0.6	0.4
